# Microbial Fermentation in Food and Beverage Industries: Innovations, Challenges, and Opportunities

**DOI:** 10.3390/foods14010114

**Published:** 2025-01-03

**Authors:** Mallari Praveen, Simone Brogi

**Affiliations:** 1 Department of Research and Development, Academy of Bioelectric Meridian Massage Australia (ABMMA), Noosaville, QLD 4566, Australia; mallaripraveen@abmma.com.au; 2 Department of Pharmacy, University of Pisa, Via Bonanno 6, 56126 Pisa, Italy

**Keywords:** microbial fermentation, food preservation, flavor enhancement, nutritional value, contamination

## Abstract

Microbial fermentation is a primary method by which a variety of foods and beverages are produced. The term refers to the use of microbes such as bacteria, yeasts, and molds to transform carbohydrates into different substances. Fermentation is important for preserving, enhancing flavor, and improving the nutritional quality of various perishable foods. Historical records clearly show that fermented foods and drinks, such as wine, beer, and bread, have been consumed for more than 7000 years. The main microorganisms employed were *Saccharomyces cerevisiae*, which are predominantly used in alcohol fermentation, and *Lactobacillus* in dairy and vegetable fermentation. Typical fermented foods and drinks made from yogurt, cheese, beer, wine, cider, and pickles from vegetables are examples. Although there are risks of contamination and spoilage by pathogenic and undesirable microorganisms, advanced technologies and proper control procedures can mitigate these risks. This review addresses microbial fermentation and clarifies its past importance and contribution to food preservation, flavoring, and nutrition. It systematically separates yeasts, molds, and bacteria and explains how they are used in food products such as bread, yogurt, beer, and pickles. Larger producers employ primary production methods such as the artisanal approach, which are explored along with future trends such as solid-state fermentation, the potential of biotechnology in developing new products, and sustainability in new product development. Future research and development strategies can lead to innovations in methods that improve efficiency, product range, and sustainability.

## 1. Introduction

### 1.1. Overview of Microbial Fermentation in the Food and Beverage Industries

**Figure 1:**
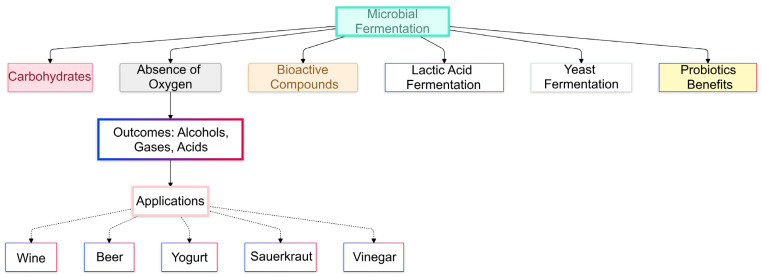
Flowchart of the fermentation process and its effects on food properties.

Microbial fermentation is a metabolic pathway where carbohydrates, e.g., sugars, grains, or any food made of the same carbohydrates, are turned into alcohols, gases, or acids in the absence of oxygen [1]. Fermentation is used in a wide variety of foods and drinks to preserve food, improve flavor, and enhance texture, in addition to providing nutritional diversity. For example, wine, beer, bread, cheese, yogurt, kimchi, sauerkraut, soy sauce, and vinegar are derived from fermentation, as shown in Figure 1 [2,3]. This trend is accompanied by the increasing importance of natural and minimally processed products in the food and beverage industries. These products hinder the development of harmful microorganisms in the food being processed through the production of inhibitory compounds such as organic acids, alcohols, carbon dioxide, bacteriocins, and others [4]. Lactic acid bacteria species convert sugars via lactic acid fermentation to lower the pH and act as a natural protective barrier against pathogenic and spoilage microbes. The concentrations of ethanol and carbon dioxide produced during yeast fermentation create an antimicrobial and anaerobic environment. Lactic acid bacteria work well on a biological basis in this bio-preservation process as bacteriocin-producing agents [5]. One of the fermentation benefits of probiotics is that the products are fortified with good bacteria, which enhance health. The result is that harmful pathogens are eliminated by a competition principle [6].

During the fermentation process, metabolite production caused by microbial activity can result in a myriad of metabolites, such as organic acids, aldehydes, alcohols, and esters, which intermix to create a wide spectrum of flavors and aromas in fermented foods [7]. For instance, ester-forming yeast and bacteria can be found in beer and wine, and amino acid conversion is the source of the savory flavor in cheese ripening and soy sauce brewing [8]. The digestive process results in the deconstruction of proteins, fats, and gases. This occurs due to chemical reactions that result in changes where solid foods change into jelly, as well as liquid–solid changes, which in turn affect mouthfeel and texture [9,10].

This comprehensive review discusses microbial fermentation, particularly its early development, food preservation effects, enhancement of food flavors, and efficiency in improving the nutritional value of food products. This research aims to study the use of yeasts, molds, and bacteria in food and beverage production, and examples include bread, yogurt, beer, and pickles. This review aims to trace artisanal and industrial approaches applied to fermentation processes, describe new tendencies, including solid-state fermentation and the biotechnology revolution, and analyze their potential to generate new approaches, diversify product portfolios, and support sustainability in the food industry.

### 1.2. The Importance of Fermentation in Food Preservation, Flavor Enhancement, and Nutritional Value

**Figure 2:**
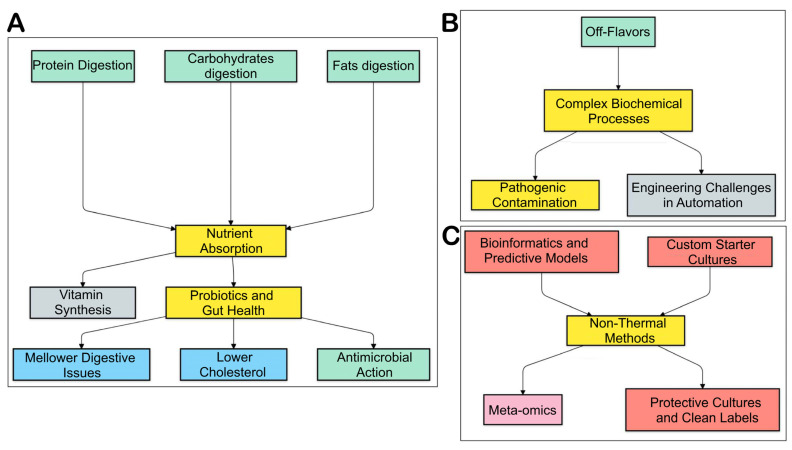
Interconnected processes and challenges in fermentation: (**A**) nutrient absorption; (**B**) probiotic health; (**C**) advanced technologies.

The process of fermentation makes proteins, carbohydrates, and fats more digestible, and nutrients in these foods are thus available to the body [6]. Moreover, lacto-fermented dairy products such as yogurt and cheese contain higher concentrations of vitamins like folate, riboflavin, and B12 because of microbial synthesis [11]. Probiotics make plant-based food products that are high in amino acids and vitamins; this is how vegan substitutes for yogurt and cheese were developed [12]. By using the good bacteria and functional foods obtained by fermentation (Figure 2A), we can have mellower digestive issues, lower cholesterol, antimicrobial actions, etc., through the modulation of the gut microbiota [6].

Even though food has been fermented for a very long time, the complex biochemical processes induced by microbial communities are still not well understood, although the development of new technology makes it possible to study the entire process better. Maintaining consistent processes between the different raw ingredients and seasonal supply chains is difficult. Pathogenic bacteria and spoilage microbes can contaminate the environment. Off-flavors could indicate poor control of the fermentation process. With the increase in automation, large-scale fermenters are struggling with engineering challenges, such as mixes, mass, and heat transfer, as shown in Figure 2B [13].

Technologies like ‘meta-omics’ have established a basis for the in-depth characterization of the microbiome, functional genes, and metabolites, greatly advancing the understanding of the molecular basis of food fermentation [3,14]. Bioinformatics, statistics, and predictive microbiology models are being used to reduce variations, and the perfection of the operational processes is shown in Figure 2C. Novel non-thermalized mechanisms such as high-pressure processing and pulsed electric field processing can make the treatment mild, allowing the maintenance of nutritional quality [14]. With protective cultures in bio-preservation, consumers can enjoy alternative clean-label products that are not chemically active. The development of custom starter cultures may provide these cultures with functional abilities to add specific sensory effects that meet the tastes of consumers.

## 2. Historical Perspective

### Historical Use and Evolution of Fermentation in Food and Beverage Production

Fermentation has been a longstanding practice since the inception of human civilization, where ancient cultures employed it for food preservation during unfavorable seasons and at ceremonial gatherings to elevate the sensory characteristics of food [15,16]. The first evidence of fermentation processes was found in stone mortars from Natufian burial sites, which belonged to a population that foraged and occasionally settled, thereby offering a 13,000-year-old archeological proof of beer production from grains [17]. Subsequently, evidence was found of the production of breads, wines, and beers dating back as far as 7000 years ago in Egypt [18]. Furthermore, chemical analysis of ancient pottery jars in China has revealed the presence of fruit products, honey, and fermented rice dating back to the seventh millennium B.C. [19]. In the early days, fermentation had two sources of microorganisms: one found in the ingredients and the other in the environment. In the course of time, empirical observation imparted particular techniques of fermentation control to ancient people, and, for instance, on the Indian peninsula, the addition of ginger to sugarcane to produce jaggery or gur became a protocol of manufacture [20].

It was only in the middle of the 19th century that Pasteur demonstrated that microbial activity was a driving factor for fermentative changes [21]. Thus, the efficiency of microorganisms in controlling and optimizing the fermentation process could be further improved by selecting from the numerous populations present in the environment. By 1890, pure yeast strains had been isolated and cultivated for winemaking fermentation [22]. The widespread introduction of microbe-killing chemicals and pasteurization methods in the early 20th century also played a role in controlling microbes during fermentation. In addition to the discovery of vitamins as growth factors, the fermentation system played a significant role in controlling yeast during this period [23].

**Figure 3:**
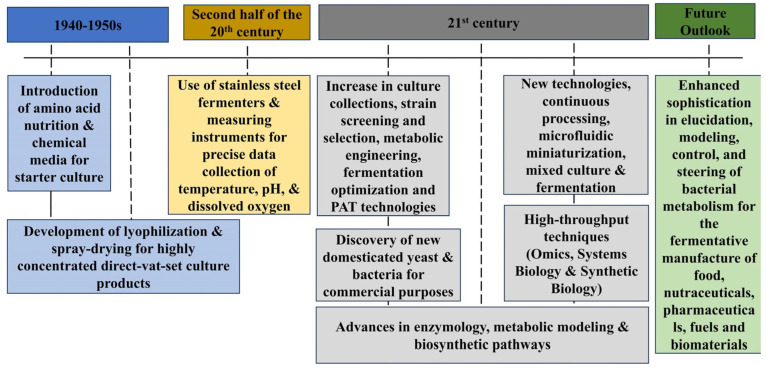
Evolution of starter culture technologies from the 1940s to present [21,22,23,24,25,26,27].

The 1940s–1950s era brought about significant modifications, such as amino acid supplementation and the development of chemical mediums to promote starter cultures [23,24,25]. Two techniques, lyophilization and spray drying, were introduced for the manufacture of highly concentrated direct-set culture products [26]. In the second part of the 20th century, a new trend was the use of stainless-steel fermenters and measuring instruments (e.g., temperature meters, pH meters, dissolved oxygen measuring devices, etc.) for obtaining precise data (Figure 3).

Currently, microbial fermentation is not only crucial in the processing of many everyday products and in the production of well-known foods and drinks. The process involves microbes, such as yeasts, bacteria, and molds, rich in carbohydrates to produce products like alcohol, acids, gases, and other compounds [1,27]. Fermentation aids in the formation of tastes and textures and, in addition, preserves perishable foodstuffs and produces useful compounds like vitamins. Some of the significant improvements in microbial fermentation are the utilization of starters to promote quality and consistency, new products with different features, including those that match customers’ tastes for artisanal or probiotic foods, and optimized bioreactor designs [28]. An important novelty is replacing wild-environment microbes with pre-adapted starter cultures. Mixed cultures, chosen with care, tend to produce a wider spectrum of flavors and volatile compounds with greater consistency and predictability that can be used in various fermented products [29]. In addition, new probiotic cultures have been discovered, and research on mixed culture fermentation is being conducted to identify new functional beverages [30]. Genetic engineering enhancements have brought about developments in genetic engineering, which in turn make it possible to carry out modifications such as increased yields, efficiency, and robustness in starter cultures [31].

**Figure 4:**
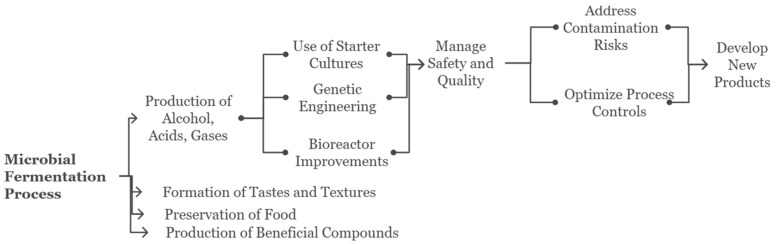
Overview of the microbial fermentation process and its applications [28,29,30,31,32,33,36,37,38,39].

Fermented foods have become very popular, but at the same time, manufacturers are striving to manage the needed safety and quality standards, as shown in Figure 4. The risks of contamination should be addressed by developing more powerful detection techniques and antimicrobial strategies [32]. Process controls must be rigorous to prevent spoilage by wild phages, bacteria, or wild yeasts/molds [33]. The other objective of manufacturers when designing a stable fermented mixture is to make it possible for them to generalize the sensory qualities that can be affected by subtle microbial community changes [34]. Another significant problem is managing the increasing cost of ingredients, regulatory issues, and consumer demand for 100% natural products. There are different methods of using fermentation to generate various foods and drinks. It is estimated that the world market for fermented products will experience a significant increase during the next few years [35]. The growing consumer appetite for healthy, environmentally friendly, and artisanal alternatives provides a chance for small innovators to excel. Potential fermentation-derived products, such as meat- and dairy-free alternatives, upcycled food ingredients, and probiotic/prebiotic formulations, are the future for consumers [29,36]. The work of producers and researchers is to discover ecological niches that differ from conventional sources of microbes, with novel bioactive properties for food processing [37].

The advantages of emerging technologies in metabolic engineering, metabolomics, and process analytics are key to advancing microbial starter culture engineering and monitoring as well as controlling the fermentation process [38]. Bioreactor improvements can be transformative in continuous processing coupled with higher yields [39]. Embracing microbial fermentation and attending to emerging technologies will lead to collective and much-needed food and beverage development [23]. The continuous improvement of food quality, safety, and sustainability, along with meeting the growing consumer demand, should be addressed in a multidisciplinary manner, encompassing food technologists, microbiologists, engineers, and nutrition scientists [40].

The latest key developments include an increase in culture collections, strain screening and selection, metabolic engineering, fermentation optimization, and PAT technologies (PAT) [41]. High-throughput culture-enabling techniques, such as omics, systems biology, and synthetic biology, are being used to improve culture improvement programs [42]. New yeasts and bacteria that have been domesticated for commercial purposes have been discovered [43]. Enzymology, metabolic modeling, and biosynthetic pathways have also been recently developed and have contributed to this multifaceted field [44]. These new technologies include continuous processing, microfluidic miniaturization, and mixed culture and fermentation [45,46]. They offer horizons not previously seen. The future beckons undeviatingly, bringing enhanced sophistication to the elucidation, modeling, control, and steering of bacterial metabolism toward the fermentative manufacture of food, nutraceuticals, pharmaceuticals, fuels, and biomaterials.

## 3. Microorganisms Used in Food and Beverage Fermentation

### 3.1. Yeasts and Molds

**Figure 5:**
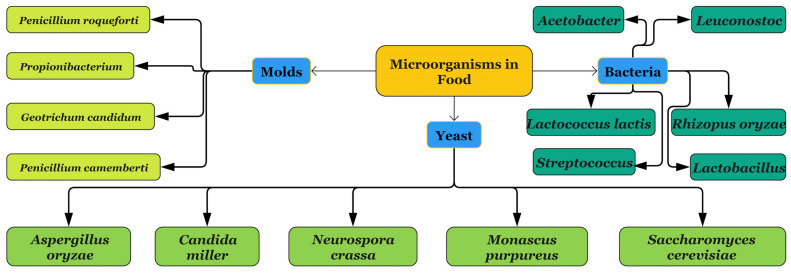
Key microorganisms in food production: molds, yeasts, and bacteria.

It is important to point out, however, that some types of yeasts and fungi are valuable for food production because of their metabolic abilities (Table 1 and Figure 5). For instance, *Saccharomyces cerevisiae* is a yeast strain that is used to produce beer, bread, wine, cider, sake, and kefir [47], and *Candida miller* is used to produce fermented products such as kombucha, sourdough, and kefir [48]. Furthermore, the role of *Aspergillus oryzae* in the production of soy sauce, miso, sake, rice vinegar, and koji cannot be ignored [49]. *Penicillium camemberti* is used to produce soft cheeses like camembert and brie, which are of great relevance [50]. Several *Rhizopus* spp., most notably *Rhizopus oryzae*, are used as the basis for tempeh and other traditional fermented products [51]. The mold *Neurospora crassa* is known as koji and is used to produce soy sauce, miso, and sake [52]. Last but not least is *Monascus purpureus*, which is the strain that produces red yeast rice, which is used in diverse Chinese recipes [53]. The production and preservation of foods are key elements for which molds serve as fundamental agents (Table 1 and Figure 5). *Aspergillus oryzae* is the main fungus used in the production of soy sauce, sake, miso, rice vinegar, and koji. *P. camemberti* is the key factor in the formation of the particular texture and taste of cheese of the types of camembert or brie [54,55], and *P. roqueforti* is the major player in the formation of blue-veined varieties such as roquefort, stilton, and gorgonzola [56]. White mold predominantly occurs on rinds due to the activity of *Geotrichum candidum*, another fungus among other natural communities, on the surface of sun-ripe cheeses and sour cream, with a substantial effect on their development and flavor [57,58]. Although mold-growing conditions are essential for developing specific textures and tastes in cultured dairy products, they can be moderated to provide the best outcomes.

### 3.2. Bacteria

Table 2 and Figure 5 illustrate the varieties of bacteria that are crucial in food fermentation (e.g., lactic acid bacteria, acetic acid bacteria), highlighting their role in the production of foods such as yogurt, cheese, sauerkraut, kimchi, pickles, and vinegar [71,72,73,74]. The genera of bacteria most cultivated are *Lactobacillus*, *Streptococcus*, *Lactococcus*, *Propionibacterium*, *Acetobacter*, and *Leuconostoc* [75]. The fermentation processes that produce yogurt, cheeses, sauerkraut, kimchi, pickles, and fermented vegetables are aided by beneficial *Lactobacillus* spp., such as *L. acidophilus, L. delbrueckii*, and *L. plantarum*, among others [76]. Another significant bacterium, *Streptococcus thermophilus*, is found in yogurt and cheese starter cultures [72]. *Lactococcus lactis* subsp. *lactis* and *cremoris* are key strains resulting in the production of cheese, buttermilk, and sour cream [54]. *P. freundenreichii* and *P. acidipropionici* are indispensable in the production of foods such as Emmental and Gruyère, whose main ingredients are milk and butter [75]. *A. aceti* and *A. pasteurianus*, members of the *Acetobacter* spp. are exploited to produce vinegar and kombucha drinks [76]. Lastly, *Leuconostoc mesenteroides* and *L. lactis* are included in seed culturing for vegetable fermentation, such as pickles, sauerkraut, and kimchi [77,78]. The controlled application of such an array of desired bacteria opens up food preservation alternatives on the one hand and enhances flavors, textures, and nutritional value on the other hand.

## 4. Types of Fermented Food and Beverage Products

Fermented foods and drinks play significant roles in various human diets, and several experimental studies have demonstrated their potential benefits to human health. Research from various continents has established correlations between the microbes found in specific fermented foods, such as agave fructans, kefir, yeast, kombucha, cheeses, and vegetables, and a range of health benefits, including weight management, reduced risk of cardiovascular and gastrointestinal diseases, antidiabetic effects, relief from constipation, improved glucose and lipid levels, enhanced immunological functions, anticancer properties, and decreased mortality rates, promoting human health [88,89,90,91]. Further investigation is required to understand the roles of fermented traditional foods and beverages in the prevention or management of these conditions [92,93]. Food fermentation is ensured by the biochemical effects of microbiological entities. Such microbiological entities include bacteria, yeasts, and molds (Table 3). Common fermented dairy products include yogurt, which is created using specific bacteria, the most common of which are *Lactobacillus bulgaricus* and *Streptococcus thermophilus* [94]. Another fermented dairy product is cheese, which can be produced with the aid of several specific bacteria species such as *Lactobacillus* spp. and molds like *Penicillium* spp. Yeast is the main protagonist in producing beer and wine [95]. For example, beer is made from *Saccharomyces cerevisiae* brewer’s yeast, while wine employs both *Saccharomyces cerevisiae* wine yeast and other types of wild yeasts and bacteria. Cider, on the other hand, is produced using corresponding wild yeasts and bacteria [96]. During the fermentation of salami, lactic acid bacteria (*Lactobacillus* spp.) are present in addition to molds like *Penicillium* spp. [97].

In the case of fermented fish, salt-tolerant bacteria, yeasts, and molds are used [98]. Fermented vegetables are displayed as sauerkraut [99], made from *Leuconostoc mesenteroides* bacteria, and kimchi [96], which contain *Lactobacillus plantarum*, yeasts, and molds as cultures [97]. Sourdough bread is the fermenting of grain and legume products aided by the presence of wild yeasts, like *Saccharomyces cerevisiae*, and lactic acid bacteria, such as *Lactobacillus sanfranciscensis* [100,101,102,103,104], in a process also known to produce products like tempeh, which is the result of the fermentation of soybeans by *Rhizopus oligos*. The unique tastes and preservative properties of fermented foods are brought about by microbial diversity. Recently, the food industry has faced a significant challenge in creating high-quality vegan products with improved nutritional, functional, and sensory attributes, given that 79 million people currently follow a vegan diet. Accordingly, in this scenario, researchers in the scientific community are examining natural and sustainable ingredients to create new and innovative food products [105,106]. The dairy industry places great emphasis on the ability of probiotic bacteria to remain viable throughout the production and storage of fermented milk. Recent studies have focused on incorporating microalgae into milk to enhance the viability of probiotics. Microalgae such as *Spirulina platensis* and *Chlorella vulgaris* are the most widely known varieties for their use in fermented milk products [107,108,109]. These factors impact not only the stability and functionality of probiotics in the final products but also their sensory qualities. Adding microalgae to probiotic-fermented milks and improving the viability of probiotics can enhance their functional properties. This is because they contain a wide range of nutrients and nutraceuticals and are considered “functional foods”, and they can play a crucial role to maintain the gut microbiota in healthy conditions [6,110]. Furthermore, the nutritional value of microalgae’s combination with probiotic bacteria is high and cost-effective because of the high concentration of lactic acid bacteria. Future research may incorporate microalgae varieties into fermented milk and yogurt products to enhance the health benefits of these products.

**Table 3:** The diversity of fermented food and beverage products and the various microorganisms involved in their fermentation processes, each contribute to the unique characteristics of the final products.

Fermented Product	Examples	Microorganisms Used	Refs.
Dairy Products	Yogurt	*Lactobacillus bulgaricus*, *Streptococcus thermophilus*	[94]
	Cheese	Various lactic acid bacteria (e.g., *Lactobacillus* spp.), molds (e.g., *Penicillium* spp.)	[95]
Fermented beverages	Beer	*Saccharomyces cerevisiae* (brewer’s yeast), various bacteria for sour beer styles	[96]
	Wine	*Saccharomyces cerevisiae* (wine yeast), various wild yeasts and bacteria	
	Cider	Various wild yeasts and bacteria	
	Milk	*Spirulina platensis* and *Chlorella vulgaris*	[107,108,109]
Fermented meats & fish	Salami	Lactic acid bacteria (e.g., *Lactobacillus* spp.), molds (e.g., *Penicillium* spp.)	[97,99]
	Fermented fish (e.g., Surströmming)	Various salt-tolerant bacteria, yeasts, and molds	[98]
Fermented vegetables	Sauerkraut	Lactic acid bacteria (e.g., *Leuconostoc mesenteroides*)	[96,101]
	Kimchi	Lactic acid bacteria (e.g., *Lactobacillus plantarum*), yeasts, and molds	
Fermented grains & legumes	Bread (sourdough)	Wild yeast (e.g., *Saccharomyces cerevisiae*), lactic acid bacteria (e.g., *Lactobacillus sanfranciscensis*)	[97,102]
	Tempeh (fermented soybeans)	*Rhizopus oligosporus*	[100]

## 5. Challenges in Fermented Food and Beverage Production

### 5.1. Microbial Contamination and Spoilage

**Figure 6:**
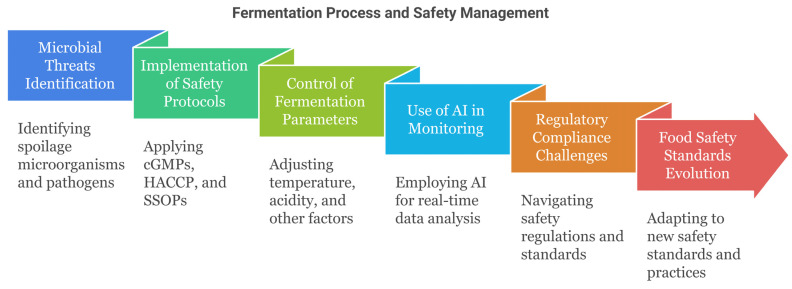
Fermentation issues during food and beverage production.

Spoilage microorganisms and pathogens are persistent threats to the production of fermented foods and beverages. Sometimes, souring and biogenesis of yeasts and wild lactic acid bacteria lead to unpleasant tastes, gas pockets, discoloration, and other defects during the aging process [111,112,113]. Another concern regarding the manufacture of fermented foods is that it is possible for pathogens like *Listeria monocytogenes*, *Salmonella* spp., pathogenic *Escherichia coli*, and mycotoxigenic fungi to contaminate raw ingredients or processing equipment and survive some of the fermentation controls shown in Figure 6. Finding, identifying, and tracking the sources of such microbes can be difficult owing to complex and varying microbial ecosystems during fermentation process production [93]. The implementation of standard cGMPs, HACCP programs, SSOPs, and ingredient/material testing ensures that risks to food safety are very much controlled, even though sporadic contamination cases cannot be fully prevented [22].

### 5.2. Control of Fermentation Parameters

In addition, accurate control of parameters such as temperature, acidity, dissolved oxygen, redox potential, and duration is vital for achieving related biochemical transformations, desired functional properties, and appetizing tastes in fermented products. Nevertheless, the quality of ingredients plays a significant role in the variability of microbial starter culture performance, as well as the equipment of the production unit, which may limit the ability to control the fermentation kinetics tightly [114]. Online monitoring systems and software for real-time data analysis based on artificial intelligence (AI) algorithms are currently used to control parameters to preserve production and food quality and safety; however, despite their high potential, these strategies are not used at all industrial levels [115]. The absence of reinforcement between fermentation conditions leads to product defects or failure.

### 5.3. Regulatory Considerations

Regulatory requirements give attention to safety but create problems for which validation activities for microbial inactivation or testing protocols, specialized sanitary equipment, and different facilities for the fermentation of products with diverse food safety risks are necessary [116]. The use of histamine-producing lactic acid bacteria strains, which are often involved in kimchi and cheese production, is limited; therefore, achieving the desired flavors, textures, and other attributes in some fermented foods is challenging [117]. The regulations can include only ingredients that shall be used, additives that can be used, and production processes that are allowed. Evaluating safety and complying with regulatory requirements for innovative fermented foods and manifold production techniques surely requires a great deal of time and money as well [118].

### 5.4. Food Safety Standards

As food safety authorities endeavor to improve the standards for risk reduction, we expect further risk reduction. For instance, the U.S. Food Safety Modernization Act, which is currently focusing on the environmental monitoring of *Listeria*, has encouraged fermented food and beverage companies to either buy new environmental sampling programs or add new features to existing systems [28]. These actions require the development of microbiological expertise and specialized laboratories [114]. New disclosure standards for histamine, biogenic amines, and food allergens may also prompt the review of the current fermentation processes [28,119]. In addition to the increased production costs and complexity of management, safety standards and fitness objectives are also subject to change.

## 6. Opportunities for Innovation and Growth

Existing and evolving consumer preferences trigger new ideas and growth opportunities for the fermented food and beverage sector. Consumers are on the hunt for products they view as natural and whole, free from artificial ingredients, handcrafted, and ecologically safe [120]. This process creates and improves savory tastes, textures, nutritional benefits, and shelf life. The population expects all these qualities in modern food products.

### 6.1. Market Trends and Consumer Preferences

Probiotics and probiotic foods have become increasingly popular among the public for their gut health. According to an expanded forecast, the global probiotics market will expand from $52.1 billion in 2021 to $79.4 billion in 2028 [121]. Some foods that promote healthy gastrointestinal bacteria include yogurt, kefir, kimchi, kombucha, and other fermented foods. Innovation has the power to tailor probiotic strains and the functions of products to health interests that consumers have, such as immunity, digestion, cognition, and weight management [122].

**Figure 7:**
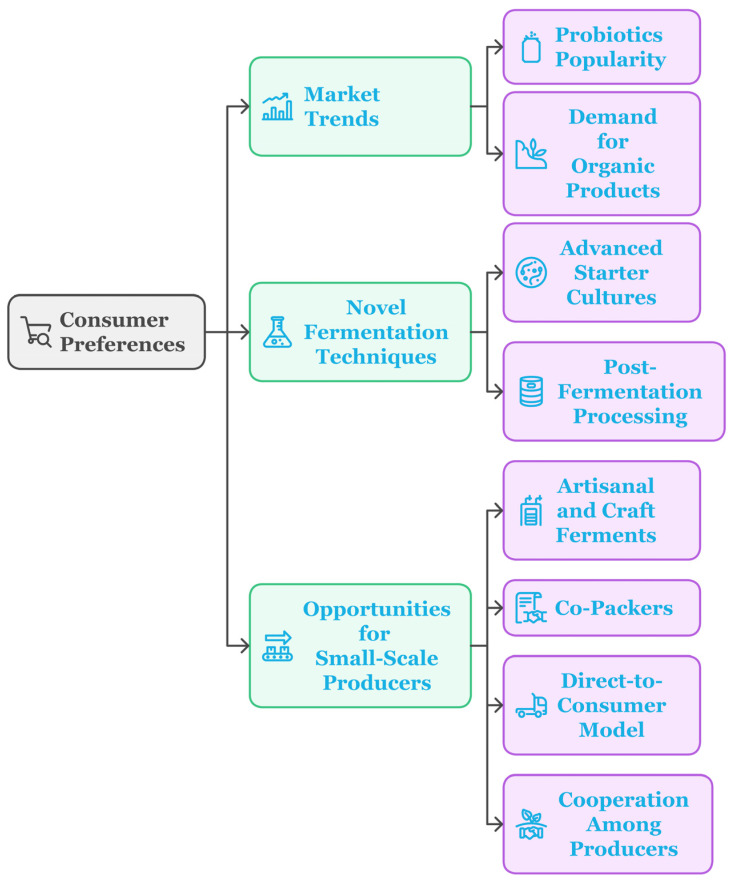
Consumer preferences driving innovations and opportunities in fermentation processes.

Consumers tend to trust more in the organic aspect and expect ingredients to be listed on the packaging with a clear indication of the methodology behind the production process [123]. Consequently, there are niches for emerging small-scale manufacturers of craft kombuchas, pickles, sauerkraut, ginger beers, and sourdoughs, which are marketed as local and transparent (see Figure 7). Traditional methods of making and increasing the quality of fermented foods also offer opportunities for innovation.

### 6.2. Novel Fermentation Techniques

Through biotechnological innovation, automation, monitoring, and processing techniques, fermentation and product differentiation can be upgraded. Advanced starter cultures, fermentation microbial enzymes, and novel fermentation conditions enable the development of new flavors and aromas. Organic acids, ethanol, and specific nutritional outcomes can be regulated through innovations like mixed-culture fermentation or the metabolic engineering of microbes [124].

Novel post-fermentation processing technologies indeed give rise to opportunities. Unlike traditional thermal processes such as heating and cooling, new technologies such as ultrasound, high-pressure processing, and pulsed electric fields can help to maintain the freshness and stability of products with heat sensitivity [123]. Packaging innovations for solid and liquid films also inhibit spoilage after fermentation is complete [125].

### 6.3. Opportunities for Small-Scale Producers

The growing interest in artisanal and craft ferments has been the main driver of microbial fermentation, which is an important part of small businesses and start-ups [126]. The low equipment necessity and the likelihood of differentiation through exclusive fermentation processes are some of the advantages of relatively small manufacturers over large entities. Co-packers are the biggest reason behind the twofold expansion of small brands’ production capacity as well as maintaining artisanal methods [127].

Minor producers will have the advantage of the long shelf life imparted on products with the fermentation process and the ability to join local food movements. The direct-to-consumer model, presented through farmers’ markets, arrangements, and online retail, connects distribution channels for start-ups [128]. Cooperation among regional farmers cum producers facilitates the transference of knowledge, resource sharing, and marketing. Ongoing innovation will present products with original fermented tastes, thus separating artisanal producers from others. This will ensure their competitive edge and longevity.

Over the past decade, there has been a growing interest in developing innovative fermentation technologies to add value to the food industry by using processing by-products from vegetable and animal sources. The objective of industrial fermentation is to convert by-products into more nutritious substances or to create bioactive compounds and to efficiently manufacture pigments, enzymes, fertilizers, and fuels sourced from biological materials [129,130,131,132]. In fact, the fermentation of various by-products from vegetables and animal sources, including soya, rice, barley, citrus, poultry, meat, and fish, has been reported using different microorganisms [114,133]. This indicates the potential for developing new circular economy strategies through the fermentation of agrofeed residues by lactic acid bacteria, either on their own or in conjunction with other microorganisms. Fermentation strategies involving lactic acid bacteria have been supplemented by the use of other fermentative bacteria to valorize vegetable by-products, such as various species of *Clostridium* and *Bacillus* [134]. For example, soya by-products underwent solid-state fermentation at temperatures ranging from 30 °C to 47 °C, often using *Aspergillus niger* and *Bacillus* species, or yeast at temperatures between 20 °C and 28 °C [135]. Barley bran and brewing waste were mainly inoculated by *Aspergillus*, *Trichoderma*, and lactic acid bacteria species. A further example involved the edible fungus Neurospora intermedia, which uses bread waste as a feedstock in a solid-state fermentation process to manufacture a protein-enriched food product, demonstrating the potential for more environmentally friendly management of discarded bread [130]. Interestingly, the possibility of using vegetable and fruit waste to generate biofuels, particularly bioethanol, has also been highlighted. Vegetable wastes high in cellulose, hemicelluloses, and lignin are used to produce second-generation bioethanol [136].

A potential opportunity exists to utilize food industry waste as animal feed. This development is quite promising, as it would yield both environmental and social advantages alongside lowering animal production expenses [134,137]. Studies have found that fish by-products, including non-edible components like heads, internal organs, skin, and bones, of *Dicentrarchus labrax* can be fermented by specific strains of the microorganisms *S. cerevisiae* and *Lactobacillus reuteri* supplemented with lemon peel as a filler. In this process, fish waste is converted into high-protein supplements for aquaculture feed formulations. The end product of fermentation contained minimal levels of spoilage-causing microorganisms but was abundant in beneficial microorganisms with lipid and protein compositions suitable for use in aquaculture feed [137].

Animal by-products originating in the slaughterhouses of large animals and poultry include meat and bone offal, blood, intestines, poultry heads and feet, fat, feathers, bowels, hooves, horns, animal hair, stomachs, intestine and rumen content, and the carcasses of animals disqualified from human uses. Accordingly, for example, the use of different by-products has been attempted in different type of pet foods, and for feeding other animals besides pet dogs, since these by-products are rich in protein, fats, vitamins, and minerals [114,138].

## 7. Health Benefits of Fermented Foods and Beverages: An Overview

Over the past few years, a rapidly growing trend toward minimally processed foods has elevated the appeal of fermented foods as nutritionally and functionally advantageous, becoming integral components of many diets. According to the growing interest in fermented foods, scientists are seeking to establish the effects of these foods on human health and to identify the most effective ways in which they can be incorporated into one’s diet [91,139]. For this reason, the fermentation process and its end products have received substantial scientific interest, and the microorganisms that contribute to the fermentation process have recently been associated with many health benefits, although some studies did not confirm this relationship. For example, during fermentation, lactic acid bacteria synthesize vitamins, produce micronutrients, and produce biologically active peptides that are also well known for their health benefits (i.e., conjugated linoleic acids that demonstrate significant influence in lowering blood pressure; bacteriocins that exhibit antimicrobial effects; exopolysaccharides that show prebiotic properties, etc.) [83]. Accordingly, in this section, we briefly discuss some studies that have highlighted the beneficial effects of fermented foods and beverages. In this regard, numerous dietary studies have been conducted on the impact of fermented product consumption on cardiovascular diseases, type 2 diabetes, and weight management. In particular, a metanalysis conducted by Chen and colleagues analyzed the relationship between different types of dairy products and the risk of type 2 diabetes in 14 prospective cohorts with 459,790 participants. Results showed that higher intake of yogurt was associated with a reduced risk of type 2 diabetes, whereas other dairy foods and total consumption of dairy were not appreciably associated with the incidence of type 2 diabetes [140]. Several studies have indicated that the consumption of fermented foods such as yogurt and cheese can have a positive impact on reducing the risk of developing cardiovascular diseases [90,141,142]. The consumption of dairy products has been suggested to ameliorate the characteristics of metabolic syndrome, which encompasses a cluster of risk factors including dyslipidemia, insulin resistance, increased blood pressure, and abdominal obesity, which together markedly increase the risk of diabetes and cardiovascular diseases [142]. A recent metanalysis conducted by Zhang and colleagues analyzed data from the years 1980 to 2018 on the association between fermented dairy food intake and cardiovascular risk. The researchers selected 10 studies that met the inclusion criteria. In total, 385,122 participants, 1392 myocardial infarction cases, 4490 coronary heart disease, 7078 stroke, and 51,707 uncategorized cardiovascular cases were considered. Overall, statistical evidence of significantly decreased cardiovascular risk was found to be associated with the intake of fermented dairy foods. In a subgroup analysis, cheese and yogurt consumption was associated with decreased cardiovascular risk, indicating that fermented dairy food intake was associated with decreased cardiovascular risk [143]. In general, increased consumption of yogurt, kefir, and other fermented foods has been driven, in part, by the health benefits that these products confer [141]. The fermented product kefir is defined as a natural probiotic with healthy effects because of the presence of a large number of microorganisms and their microbial interactions, and the presence of bioactive compounds (i.e., organic acids, CO_2_, H_2_O_2_, ethanol, bioactive peptides, exopolysaccharides (kefiran), and bacteriocins). Accordingly, several studies have shown that kefir and its constituents have an antimicrobial effect against numerous bacteria, including *E. coli*, *L. monocytogenes*, *Salmonella Typhimurium*, *Candida albicans*, *Shigella sonnei*, and *Staphylococcus aureus* [144,145,146], show antitumor and immunomodulatory activity [147,148], improve lactose digestion [149], and reduce the risk of cardiovascular diseases, among others [64].

The fermented food kombucha is a noteworthy source of B complex vitamins, polyphenols, and organic acids (mainly acetic acid). Nowadays, kombucha tends to be prepared with some other plant species, which, therefore, leads to variations in its composition. Pre-clinical studies conducted on kombucha revealed that it has desired bioactivities such as antimicrobial, antioxidant, hepatoprotective, antihypercholestorelomic, anticancer, anti-inflammatory, etc. Unfortunately, only a few clinical studies have been reported to confirm the preclinical evidence and valuable biological effects on human health [65,150,151]. Interestingly, antidiabetic and antiobesity effects were associated with kimchi consumption [83]. In a study in which 21 participants with prediabetes were administered fresh or fermented kimchi for 8 weeks, it was observed that consumption of both types of kimchi significantly decreased body weight, body mass index, and waist circumference. Fermented kimchi decreased insulin resistance and increased insulin sensitivity. Systolic and diastolic blood pressures were significantly decreased in the fermented kimchi group. The percentage of participants with improved glucose tolerance was 9.5% and 33.3% in the fresh and fermented kimchi groups, respectively. Results indicated that consumption of kimchi has a beneficial impact on human health [152].

Buttermilk has been extensively investigated for its beneficial effects on human health. Conway and colleagues evaluated the effects of buttermilk on blood pressure, markers of the renin–angiotensin–aldosterone (RAS) system, plasma lipids, and markers of cholesterol (C) homeostasis in a randomized, double-blind, placebo-controlled, crossover study. Men and women (N = 34) with normal blood pressure were supplemented with 45 g/die of buttermilk and with 45 g/die of a macro-/micronutrient-matched placebo in random order (4 weeks for each diet). Buttermilk consumption significantly reduced systolic blood pressure (−2.6 mm Hg; *p* = 0.009), mean arterial blood pressure (−1.7 mm Hg; *p* = 0.015), and plasma levels of the angiotensin I-converting enzyme (−10.9%; *p* = 0.003) compared with the placebo, but had no effect on plasma concentrations of angiotensin II and aldosterone [153]. Furthermore, consumption of buttermilk reduced serum cholesterol (−3.1%, *p* = 0.019), LDL-C (−3.1%, *p* = 0.057) and triacylglycerol (−10.7%, *p* = 0.007). Buttermilk consumption increased plasma lathosterol concentrations (+12.1%, *p* = 0.001) [154]. Other studies have indicated the strong antioxidant profile of buttermilk, acting as a scavenger for both hydroxyl and peroxyl radicals and a metal chelating agent in vitro [155,156]. Buttermilk was investigated as a potential anticancer agent and demonstrated the capability of inducing caspase-independent cell death and attenuating Wnt, Akt, and ERK signaling in a cell line of colon cancer [157].

The famous red yeast rice is used as an antihypercholestorelomic agent. This fermented product, due to the presence of monacolin K, is able to inhibit 3-hydroxy-3-methylglutaryl coenzyme A reductase, leading to a block in cholesterol biosynthesis. It is considered the most effective cholesterol-lowering nutraceutical on the market because its efficacy has been assessed in several clinical trials [158].

Dairy products and fermented foods are associated with maintained cognitive function. Camembert cheese has also been shown to enhance cognitive function in vivo. Oleamide, derived from the fermentation of white mold, is a candidate active component that is expected to improve cognitive function and sleep conditions. Sasaki and colleagues conducted a multi-arm randomized, double-blind, placebo-controlled trial (UMIN-CTR UMIN000048084) to investigate whether milk-based cultures of white mold (MCW) and oleamide could improve cognitive function and sleep state clinically. The participants included 60 healthy Japanese (50–75 years old) who were aware of their cognitive decline and were randomly and equally divided into three groups. Participants took either MCW (equivalent to 60 μg/day of oleamide), 60 μg/day of oleamide, or placebo capsules for 12 weeks. Serum BDNF and cognitive function were the primary outcomes, whereas MCI was the secondary outcome. Furthermore, sleep status before and after treatment was assessed using the Japanese version of the PSQI (PSQI -J). The placebo group showed a negative rate of change in serum BDNF (−10.5 ± 19.7%), whereas the MCW and oleamide groups showed positive changes (2.0 ± 27.1% and 1.3 ± 13.5%, respectively). Cognitive function increased after 12 weeks in all groups of intervention. Results suggest that MCW and its component, oleamide, are safe and contribute to maintaining cognitive functions, particularly short-term and working memory, and improving sleep state [159].

Fermented foods can also have positive effects on the gut microbiome as mentioned above [83]. Kefir, a fermented milk product, has significant beneficial effects on the gut microbiota, increasing the concentrations of *Lactobacillus*, *Lactococcus*, and *Bifidobacteria* [160]. Short-term yogurt consumption for 42 days increased intestinal *Lactobacillus* [161]. A small study found that tempeh consumption increased *Akkermansia muciniphila* and immunoglobulin A, a molecule involved in the intestinal immune response [162]. Pickles are often prepared by fermentation using lactic acid bacteria that act as probiotics [82,163]. Some lactic acid bacteria strains that are part of the sourdough starter are considered probiotics that have great potential for improving gastrointestinal health [104].

According to the evidence in the literature, fermented foods and beverages could have great potential in producing beneficial effects on human health. However, the number of trials is not yet sufficient to guarantee a clear vision of the positive impact of fermented products on the dietary regimen of individuals. Further studies are required to confirm these encouraging results.

## 8. Future Directions and Research Opportunities

Among the key innovations in the field of food fermentation, we can find progress in fermentation technologies that aim to increase efficiency, expand the product range, and promote sustainability. An interesting field is solid-state fermentation, which involves the presence of microorganisms on moist non-flowing substrates to generate novel compounds, including enzymes and bioactive metabolites [38]. Major research projects would be centered around investigating factors such as temperature, moisture content, and air exposure that might be vital to mass production. Innovations in bioreactor designs are currently underway to enable the production of volumetrically equivalent solid-state fermentation with increased efficiency [164]. In this regard, a continuous fermentation system that allows continuous feeding of substrates and production removal has recently become a focus of attention. The advantages of batch processes include higher productivity, shorter turnaround times, and data that are tested for the quality of the product every time it is manufactured. While a constant bioreactor calls for fully equipped monitoring and control systems for optimal long-term performance, it also exhausts materials quickly and often leads to environmental pollution [165]. Metabolic engineering and synthetic biology are currently supported by increasingly powerful tools for enhancing fermentation strains. In-route metabolic transformation, adaptive evolution, and genome editing can alter the flux of metabolites toward target compounds or expand the utilization of the substrate [166]. With microbiota engineering, the objective is to modify microbial community ecosystems to affect population-level metabolic functions. This could make fermentation more efficient by producing culture-specific products, as well as by making the process more robust than when it is single culture based [167].

The concept of biorefining to get the maximum out of feedstock is in line with the idea of a “circular economy” that is still evolving. Through these biorefining applications in the fermentation process, the scopes of usable substrates can be expanded, useful co-products generated, and waste reduced [168]. Muscle health maintenance is a complex process, requiring advanced diagnostic techniques like metabolomics, proteomics, and Raman spectroscopy, to identify the processes occurring. In addition to machine learning-based algorithms that provide feedback or model predictive control, these techniques enable real-time optimization and quality control. Because of digitalization, smart factories manage work related to Industry 4.0 technology through automation, predictive analytics, and adaptive decision-making [169]. Sustainability is another area that would be pertinent to research on renewable inputs, green solvents, and low ecological impacts [170]. Computational simulations can solve multi-layered system problems in fermentation processes via multiscale modeling, which gives better system control and predictability when working at different scales [171].

## 9. Conclusions

Sustainable bioprocessing via microbial fermentation has been widely used in food and beverage production for several millennia. They offer several advantages, including extended shelf life, accentuating taste, and desirable nutritional qualities. However, recent improvements in fermentation technology, such as the development of microbial strains, new starter cultures, and advanced control systems, have expanded the application range of this technology. These improvements meet both old and new needs as consumers transition to natural and minimally processed foods and foods containing probiotics. However, research gaps still exist after these advancements. The communication between various microbes involved in fermentative processes remains poorly understood, especially when a mixture of species is involved. Furthermore, ingredient improvements in the integration of new biotechnologies and omics tools allow for developing accelerated microbial strains. These innovations are significant for fermenting foods of better quality, longer shelf life, and enhanced safety characteristics. Potential future uses are endless: solid-state fermentation can be used to produce new bioactive compounds and enzymes, improving food flavor. Moreover, fermentation technologies bear the potential to contribute to sustainability concerns because they afford innovative approaches to waste utilization and environmentally friendly product generation. Small-scale producers and start-ups should leverage these advancements to embrace artisan and craft fermented food products to meet the increasing demand for better and diverse food products. In this way, existing challenges have been identified and solved, and the fermentation industry has engaged in the further development of interdisciplinary study to create safer, more sustainable, and more culturally appropriate fermented products, thus ensuring the availability and quality of food and beverages worldwide.

## Figures and Tables

**Table 1:** Main yeast and fungi species used in food and beverage fermentation.

Organism	Applications	Origin/Region of Consumption	Refs.
*Saccharomyces cerevisiae* (Baker’s yeast)	Bread Beer Wine Cider Sake Kefir	China/Worldwide China/Worldwide France/Worldwide Egypt/Western Europe, Commonwealth, USA China/Asia, USA Caucasus/ Eastern Europe, Russia, Asia	[47,59,60,61,62,63,64]
*Candida* spp. (*Candida miller*)	Kombucha Sourdough Kefir	China/Asia, Russia, Eastern Europe, Germany Egypt/Western Europe, USA Caucasus/ Eastern Europe, Russia, Asia	[48,65]
*Aspergillus oryzae*	Soy sauce Miso Sake Rice vinegar Koji	China/Asia, USA China/Asia China/Asia, USA China/Asia China/Asia	[49,54,66,67,68]
*Penicillium camemberti*	Cheese ripening (e.g., Camembert, Brie)	Normandy (France)/Western Europe	[50,55]
*Rhizopus* spp. (*Rhizopus oryzae*)	Tempeh, traditional fermented foods	Indonesia/Southeastern Asia	[51,69]
*Neurospora crassa*	Koji for soy sauce Miso Sake	China/Asia China/Asia China/Asia, USA	[52]
*Penicillium roqueforti*	Blue cheese: Roquefort Stilton Gorgonzola	France/Southwestern Europe England/UK, Northern Europe Italy/Europe, USA	[56,70]
*Penicillium candidum*	White mold on cheese surfaces	Western Europe	[57,58]
*Geotrichum candidum*	Surface ripened cheeses Sour cream	Northern Europe	[57,58]
*Monascus purpureus*	Red yeast rice (used in traditional Chinese medicine and cuisine)	China/Asia, Europe, USA	[53]

**Table 2:** Bacteria species used in food and beverage fermentation.

Organism	Applications	Origin/Country of Consumption	Refs.
*Lactobacillus acidophilus* *Lactobacillus delbrueckii*	Yogurt Cheese Sauerkraut Kimchi Pickles	Turkey/Europe, Asia, Southern America, USA Northern Europe/Worldwide China/Eastern Europe, Asia, USA Korea/Asia, USA, Europe Mesopotamia/Asia, Eastern Europe, USA	[76,79,80,81,82]
*Lactobacillus plantarum*	Kimchi Sauerkraut Fermented vegetables	Korea/Asia, USA, Europe China/Eastern Europe, Asia, USA China/Eastern and Northern Europe, Asia, USA	[76,83]
*Streptococcus thermophilus*	Yogurt Cheese	Turkey/Europe, Asia, Southern America, USA Northern Europe/Worldwide	[72]
*Lactococcus lactis* subsp. *lactis* *Lactococcus lactis* subsp. *cremoris*	Cheese Buttermilk Sour cream	Northern Europe/Worldwide Asia/Eastern Europe, Asia, USA Kazakhstan/Eastern Europe, Central Asia, USA	[58,84,85]
*Propionibacterium freudenreichii* *Propionibacterium acidipropionici*	Swiss cheese, Emmental, Gruyère	Swiss/Europe, USA	[75]
*Acetobacter aceti* *Acetobacter pasteurianus*	Vinegar Kombucha	Egypt/ Western Europe, USA China/Asia, Russia, Eastern Europe, Germany	[65,86,87]
*Leuconostoc mesenteroides*	Pickles Sauerkraut Kimchi Fermented vegetables	Mesopotamia/Asia, Eastern Europe, USA China/Eastern Europe, Asia, USA Korea/Asia, USA, Europe China/Eastern and Northern Europe, Asia, USA	[77,83]
*Leuconostoc lactis*	Cheese Fermented vegetables	Northern Europe/Worldwide China/Eastern and Northern Europe, Asia, USA	[78]

## Data Availability

No new data were created or analyzed in this study. Data sharing is not applicable to this article.
